# Microscopy methods for the *in vivo* study of nanoscale nuclear organization

**DOI:** 10.1042/BST20240629

**Published:** 2025-02-04

**Authors:** Nidhi Rani Lokesh, Mark E. Pownall

**Affiliations:** Department of Biochemistry and Biophysics, University of California, San Francisco, San Francisco, CA 94158, U.S.A

**Keywords:** 3D genome organization, chromatin, nuclear organization, super-resolution microscopy

## Abstract

Eukaryotic genomes are highly compacted within the nucleus and organized into complex 3D structures across various genomic and physical scales. Organization within the nucleus plays a key role in gene regulation, both facilitating regulatory interactions to promote transcription while also enabling the silencing of other genes. Despite the functional importance of genome organization in determining cell identity and function, investigating nuclear organization across this wide range of physical scales has been challenging. Microscopy provides the opportunity for direct visualization of nuclear structures and has pioneered key discoveries in this field. Nonetheless, visualization of nanoscale structures within the nucleus, such as nucleosomes and chromatin loops, requires super-resolution imaging to go beyond the ~220 nm diffraction limit. Here, we review recent advances in imaging technology and their promise to uncover new insights into the organization of the nucleus at the nanoscale. We discuss different imaging modalities and how they have been applied to the nucleus, with a focus on super-resolution light microscopy and its application to *in vivo* systems. Finally, we conclude with our perspective on how continued technical innovations in super-resolution imaging in the nucleus will advance our understanding of genome structure and function.

## Introduction

**Figure 1:**
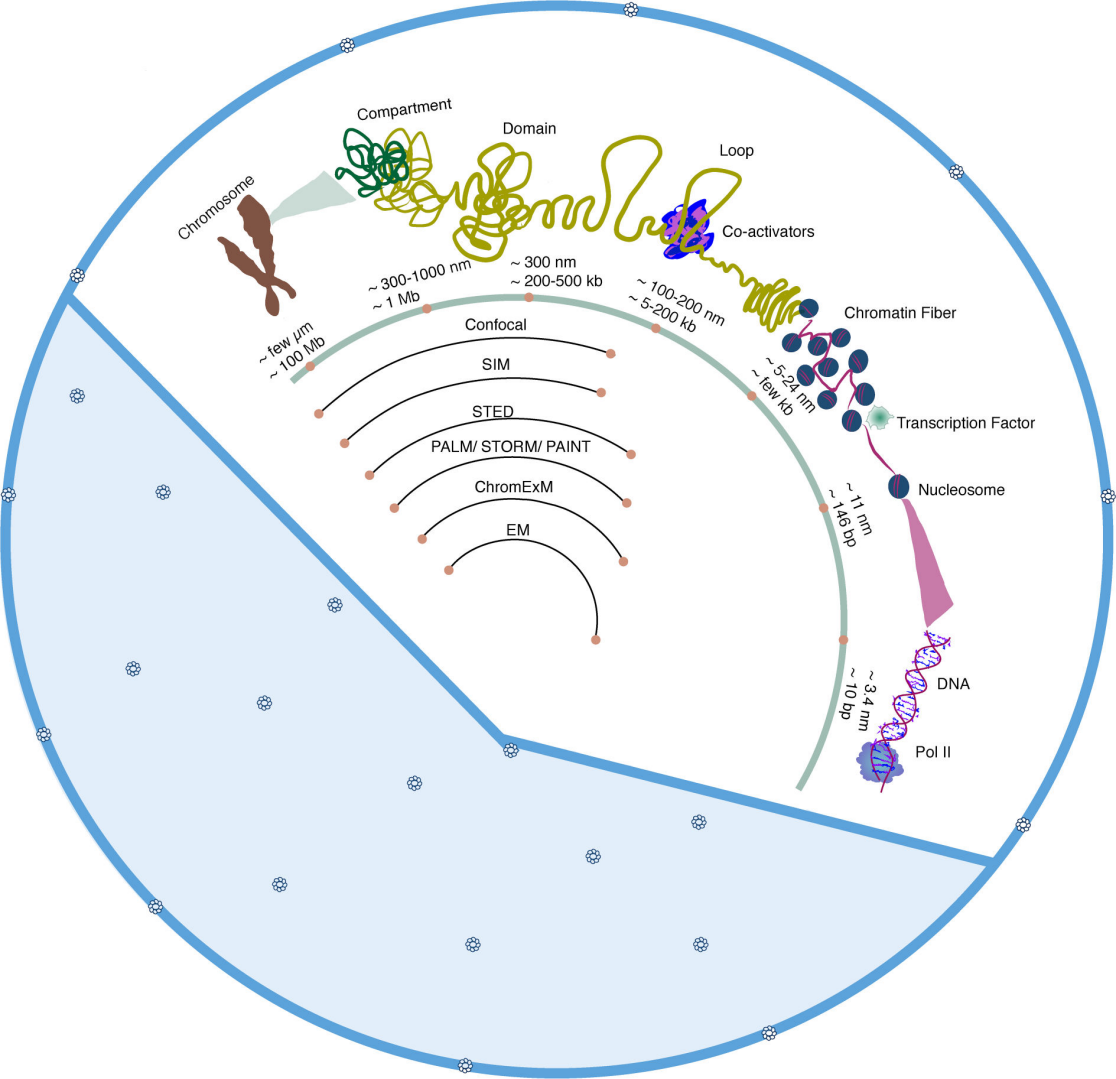
Nuclear organization across scales and methods for their visualization. Chromosomes (shown as a compact mitotic chromosome for simplicity) are formed by tightly packed compartments. Distinct compartments are made up of either active or inactive domains of loops of chromatin fibers. Chromatin fibers are made up of nucleosomes which in turn are formed when DNA wraps around histone protein complexes. Different microscopy methods are needed to visualize nuclear organization across these scales.

Eukaryotic nuclei contain a highly compartmentalized genome. The DNA is compacted across a range of scales to be contained within the nucleus. However, a vast number of regulatory elements, which control when and where genes are expressed, are distributed throughout the genome. This poses a complex challenge for regulatory factors, such as transcription factors (TFs) and co-activators, to navigate the crowded nucleoplasm and provide the specific control of gene expression that is necessary for establishing and maintaining cellular identities. It has become clear that organization within the nucleus is non-random and facilitates regulatory processes such as transcriptional control [1–4]. While many key regulatory factors and regulatory elements in the DNA have been thoroughly characterized, our understanding of how the nucleus is organized in 3D and at the nanoscale at which proteins and DNA interact remains unclear and is essential for advancing our understanding of nuclear function. However, the varying scales across which the nucleus functions, from micron-sized chromosome territories to individual molecules, have necessitated technological advancements to resolve nanoscale nuclear organization (Figure 1) [5–13]. Here, we review methods that are deepening our understanding of nuclear organization across these scales, with an emphasis on methodologies that provide a new nanoscale resolved view of the nucleus and key players within.

Techniques for assessing the nuclear organization can be broadly divided into two major categories: sequencing and imaging. Sequencing methods such as high-throughput chromosome conformation capture (Hi-C) and related techniques have enabled modeling of 3D genome folding and can infer contact probabilities of specific genomic regions [7,10,14–17]. These approaches have provided key insights into genome form and function and are extensively reviewed elsewhere [1,18–22]. In general, sequencing approaches provide insights into the population-average of the chromatin state across many cells, although single-cell 3D genomics methods present a rapidly growing field to overcome this limitation [18–20–23]. In contrast, imaging-based techniques provide direct visualization of the chromatin and other factors at single-cell resolution. This allows for a more intuitive interpretation of how the nucleus is organized and how organization relates to function using multimodal imaging. Here, we review microscopy-based approaches for studying nuclear organization, with an emphasis on those that can visualize nanoscale structures *in vivo*.

## Electron microscopy

Initially, nanoscale structures within the nucleus were identified by electron microscopy (EM) due to its high spatial resolution. However, because the common heavy metal contrasting reagents used in EM typically bind proteins rather than nucleic acids, the nucleus exhibits relatively low contrast. Thus, initial studies focused on *in vitro* reconstituted chromatin fibers, prepared chromatin spreads, or bulk contrast arising from local protein concentration in the nucleus in cultured cells. This provided early insights into the ultrastructure of chromosomes at different phases of the cell cycle, in transcriptionally active and inactive states, and the association of heterochromatin with the nuclear envelope [24–28]. Subsequently, the organization of individual RNA polymerase molecules within nuclei of cultured human adenocarcinoma cells was uncovered by combining immunogold antibody labeling with EM [29–31]. Although visualizing the chromatin itself remained a challenge, specific labeling of RNA polymerase led to the “transcription factory” model, in which RNA polymerase organizes in clusters to promote transcription.

More recently, the challenge of generating EM contrast of the DNA was overcome by chromatin electron microscopy with tomography (ChromEMT) [32]. This method has enabled specific 3D visualization of DNA at <5 nm resolution in cultured human osteosarcoma cells by coating the DNA in an osmiophilic polymer prior to imaging to enhance contrast. By visualizing chromatin *in situ*, rather than purified or reconstituted, ChromEMT uncovered that cells do not contain a 30 nm chromatin fiber, which only assembles under nonphysiological conditions *in vitro* [33]. Instead, it was observed that the chromatin is packed as a disordered fiber with diameters typically ranging from 5 to 24 nm. Similarly, chromatin imaging with scanning transmission EM (ChromSTEM) in cultured human carcinoma and fibroblast cells utilized ChromEMT staining of the DNA to identify larger-scale packing domains made up of the primary chromatin fiber [34].

It remains to be seen if ChromEMT can be combined with multimodal labeling to expand the molecular repertoire that can be visualized by this powerful technique and provide specificity beyond just the DNA. For example, combining ChromEMT with correlated light and electron microscopy (CLEM) would allow researchers to identify specific genomic loci or regulatory proteins in ChromEMT images, thereby enhancing our understanding of how different genomic regions fold and how the proteins regulating them are organized. Alternatively, combining ChromEMT and immunogold labeling could be used to pinpoint how specific chromatin modifiers associate with distinct conformations of chromatin. Because ChromEMT is limited to 250 nm thick sections of the nuclei that can be subjected to tomography, future efforts to combine ChromEMT labeling with focused ion beam scanning electron microscopy (FIB-SEM) [35–37] could enable reconstruction of the entire genome by imaging whole nuclei rather than single slices. Towards this goal, recent work has combined serial block-face scanning electron microscopy (SBF-SEM) with DNA *in situ* hybridization to visualize specific genomic sequences at 5 nm lateral resolution inside of cells by imaging across a depth of several microns instead of the thin sections used in tomography [38]. In cultured human B-lymphocytes, this revealed hundreds of unique chromatin folding structures for a 1.7-Mb region of the genome, highlighting the dynamic state of chromatin folding.

Beyond conventional EM, cryo-electron microscopy (cryo-EM) is enabling increasingly high-resolution structures to be solved, even within cells (*in situ* cryo-electron tomography; cryo-ET) [39–42]. For example, native chromatin fibers and nucleosomes within cultured human T-lymphoblasts and developing *Drosophila* embryos were recently resolved by cryo-ET, confirming previous findings that the 30 nm chromatin fiber is generally not present in cultured cells or *in vivo* [43,44]. One benefit of cryo-ET is that it does not require specific labels for the DNA or nuclear protein complexes, such as the nucleosome. This is because the resolution is sufficiently high (on the order of angstroms) that molecular species of interest can be identified based solely on their structure, often with the help of template matching or sub-tomogram averaging [45].

In general, EM-based approaches offer the highest resolution across all imaging modalities. However, it remains challenging to combine the high resolution of EM with molecular labeling techniques such as immunofluorescence or *in situ* hybridization to readily identify specific molecules of interest because fluorescence is not detected in EM, and immunogold labeling requires permeabilization and antigen retrieval steps that may alter the sample. Although some methods are bridging these gaps, techniques such as CLEM and immunogold labeling remain technically challenging and require specialized instrumentation which have limited their broad uptake by the chromatin community. In this area, light microscopy shines and is capable of resolving the identity of key molecular players within the nucleus, though generally offering slightly lower spatial resolution.

## Light microscopy

**Figure 2:**
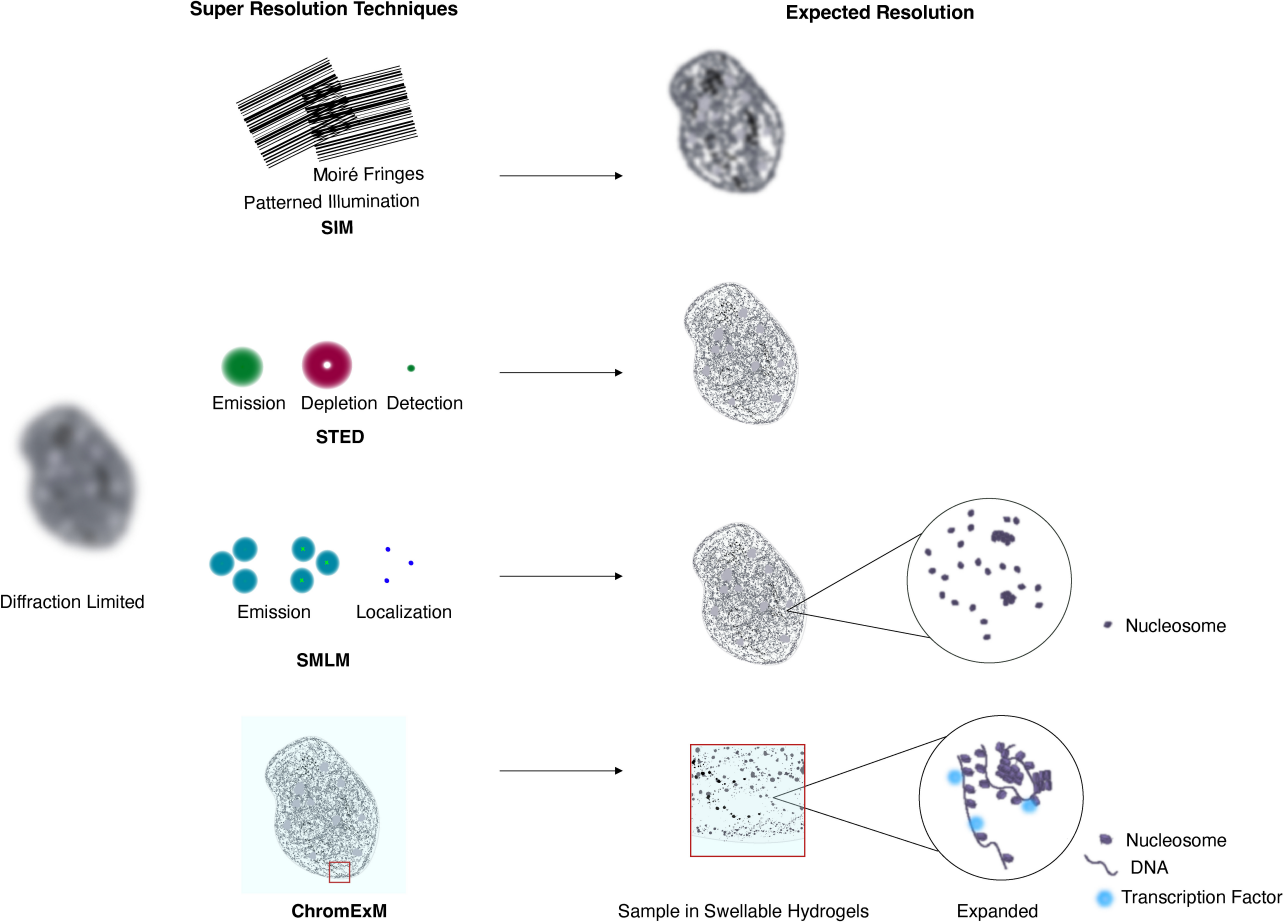
Principles of different super-resolution microscopy techniques in the nucleus. SIM: Sample is illuminated with patterned excitation light to computationally retrieve high-frequency data from the sample. STED: While an excitation beam excites fluorescent molecules, a donut-shaped depletion beam pushes the emitters back to a dark state. This effectively reduces the apparent size of the emitters. This process is repeated and fluorescent signals are used to reconstruct the sample image. SMLM: A small portion of fluorophores are activated at a time. Their positions are recorded using diffraction-limited imaging and their precise localization is identified computationally. The process is repeated iteratively to acquire data to create a high-resolution image. ChromExM: Samples are embedded in a series of swellable hydrogels and physically enlarged 15x to allow resolution of fine-scale features that would otherwise not be detected due to the diffraction limit.

While EM offers unparalleled resolution of chromatin, light microscopy excels in its labeling capabilities. However, the resolution of light microscopy is limited by the wavelength of light to a typical resolution of ~200–400 nm (depending on the numerical aperture of the imaging objective and emission wavelength being imaged) due to the diffraction limit. This has posed a challenge in assessing nanoscale nuclear organization by light microscopy despite diverse labeling capabilities. Thus, to assess nanoscale organization, it is necessary to surpass the diffraction limit with super-resolution imaging. Super-resolution microscopy techniques generally overcome the diffraction limit of light by manipulating the excitation or emission properties of fluorescent molecules (e.g., stimulated emission depletion microscopy (STED)) [46], using structured illumination (e.g., structured illumination microscopy (SIM)) [47], or temporally separating the fluorescence of nearby molecules to enable single-molecule localization with nanometer precision (e.g., stochastic optical reconstruction microscopy (STORM) [48]; photoactivated localization microscopy (PALM) [49]). These methods provide significantly higher resolution than conventional microscopy and are capable of resolving nuclear organization at the nanoscale (Figure 2). Because the principles of super-resolution imaging are reviewed in detail elsewhere [50–56], we will focus primarily on their application to the nucleus.

### Single-molecule live imaging

Live-cell imaging enables visualization of dynamic protein–protein and protein–chromatin associations at single-molecule resolution, often implementing PALM or sparse labeling of Halo, SNAP, or CLIP tagged proteins [49,57–63]. In these techniques, only a small portion of molecules are imaged such that individual fluorescent molecules are separated by distances larger than the diffraction limit and can thus be individually resolved. Nonetheless, these techniques have provided deep insights into nuclear function at the nanoscale. In cultured mammalian cells, and *Drosophila* and zebrafish embryos, single-molecule live imaging has shown that TFs tend to have short residence times on the chromatin, on the order of a few seconds [64–70]. By combining single-molecule live imaging with transcriptional reporters, researchers have gained new insights into gene regulation, uncovering how Pol II clusters dynamically activate transcription via association with co-activators [71–74]. Similarly, single-molecule tracking has demonstrated that architectural proteins, such as cohesin and CTCF, which promote DNA looping, have considerably longer residence times on chromatin, on the order of minutes in both cultured mammalian cells and living zebrafish embryos [75,76]. Additional live imaging experiments labeling individual CTCF sites on the genome in mouse embryonic stem cells have shown that chromatin loops are formed transiently, with the fully looped state being relatively rare [77,78]. Taken together, these studies highlight the power of live imaging to uncover dynamic nanoscale interactions in the nucleus and provide key insights into the regulation of genome architecture and gene expression.

### Localization-based super-resolution microscopy

Relying on similar principles that enable single-molecule live imaging, localization-based super-resolution microscopy (often referred to as single-molecule localization microscopy (SMLM)) reaches ~20–30 nm lateral resolution in fixed samples and resolves individual molecules of interest in the nucleus (Figure 2). In fixed cells, STORM is perhaps the most commonly used SMLM technique due to its high resolution and localization precision (~tens of nanometers). STORM relies on the ability of certain fluorescent dyes to stochastically switch between a bright and dark state. This allows for only a small portion of molecules to be in the bright state at a given time to enable single-molecule localization [48].

Multi-color STORM has been used to uncover how nucleosomes are organized into distinct clutches by assessing the spatial relationship between individual nucleosomes [79,80]. These studies in cultured human somatic cells and mouse embryonic stem cells demonstrated that chromatin fibers are formed by heterogeneous groups of nucleosomes, consistent with the observations of ChromEMT, and suggest that histone tail post-translational modifications influence local chromatin compaction. Additionally, this methodology has enabled direct visualization of how chromatin function (e.g., transcription) can relate to its local organization using multi-color STORM imaging of nucleosomes, DNA, nascent RNA, and Pol II, among other factors [81–83]. Similar work has found that DNA itself compacts into so-called “nanodomains”, consistent with the observations of nucleosome clutches [84].

A key application of STORM in the nucleus is in combination with chromatin tracing, which provides sequence-specificity when imaging the DNA at high resolution [85,86]. Chromatin tracing has been implemented in several different ways, but the underlying principles are shared. Briefly, a series of DNA fluorescence *in situ* hybridization (FISH) probes are sequentially imaged along a region of interest (typically imaging in steps of ~5–100 kb) to walk along the chromosome and precisely localize each region. Each genomic region is imaged sequentially via multiple rounds of secondary probe hybridization to allow precise localization of the centroid position of the diffraction-limited spot containing this region. This principle is analogous to that of most SMLM techniques in which the fluorescence of individual molecules, or in this case genomic regions, is temporally separated to enable super-resolution. In this way, each genomic region of interest appears as a diffraction-limited spot, and neighboring regions are imaged sequentially such that each spot is precisely localized without overlapping neighboring regions. Initial studies were performed at the scale of whole topologically associating domains (TADs) in cultured human fibroblasts and found that TADs are spatially organized into A and B compartments depending on their transcriptional activity [87]. Subsequently, chromatin tracing was combined with STORM to increase the genomic resolution to ~10–30 kb and resolved the structure of these individual segments in cultured human fibroblasts [88,89]. Other advances have extended the multiplexing abilities of chromatin tracing to the whole-genome scale using DNA multiplexed error robust FISH (DNA-MERFISH) [90]. Chromatin tracing has also been applied *in vivo*: in *Drosophila* embryos, optical reconstruction of chromatin architecture (ORCA) and high-throughput multiplexed imaging (Hi-M) have uncovered *cis*-regulatory and epigenetic interactions within the chromatin and how chromatin contacts change during early development [91,92]. In mice, ORCA has identified how 3D chromatin contacts influence limb development [93] and how specific loci are compacted in developing brain tissue [94], while Multiplexed Imaging of Nucleome Architectures (MINA) has identified how chromatin folds across scales in specific cell types within fetal liver tissue [95]. Additionally, a similar technology (epigenomic-MERFISH) has enabled multiplexed imaging of various epigenetic modifications in embryonic and adult mouse brains to begin identifying how histone modifications are spatially organized within the nucleus and relate to genome function [96]. In *Caenorhabditis elegans*, chromatin tracing has mapped homologous chromosome overlap and the timing of chromatin compartmentalization during development [97,98]. These works highlight how chromatin tracing can uncover nanoscale organization of chromatin, with sequence-specificity, *in vivo* and provide functional understanding of these structures when coupled with simultaneous protein and RNA imaging.

Another approach for generating single-molecule localizations is DNA points accumulation for imaging in nanoscale topography (DNA-PAINT). DNA-PAINT relies on the same principle as other SMLM techniques but generates individual localizations by transient hybridization of fluorophore-conjugated oligos to DNA handles attached to the target of interest (e.g., secondary antibody with a DNA handle instead of a fluorophore) [99,100]. This transient hybridization achieves temporally separated spots of signal for resolving single molecules and allows relatively straightforward multiplexing of multiple targets since the probes can be washed out. Thus, a wide variety of fluorophores can be used with DNA-PAINT as the method does not rely on the photophysics of the fluorophore itself to achieve single-molecule localization. This is in contrast to methods like STORM, which rely on the properties of the dye itself to achieve localizations. DNA-PAINT has been extended to allow nearly unlimited multiplexed 3D super-resolution imaging in cells at <15 nm lateral resolution [101,102]. While many methods are limited to multiplexing 3–4 targets at a time, this type of DNA-PAINT could enable substantial discoveries in the nucleus, such as visualizing the binding of multiple TFs, co-activators, and Pol II simultaneously at single-molecule resolution to better understand mechanisms of transcriptional activation.

One advantage of DNA-PAINT is that the localization of individual molecules is dependent on DNA binding kinetics, allowing for continued advances to increase imaging speed [101–105]. Although advances in DNA-PAINT have provided outstanding increases in throughput and multiplexing abilities at super-resolution, its application in the nucleus has faced some challenges due to the potential for DNA imaging strands to hybridize with genomic DNA, leading to nonspecific signals. Recent work has developed left-handed DNA imaging oligos, which do not hybridize with naturally occurring right-handed DNA, to improve DNA-PAINT imaging in the nucleus [106]. Future work combining left-handed DNA-PAINT with recently developed accelerated imaging and multiplexing strategies will enable substantial advances in our understanding of nuclear organization.

### Structured illumination microscopy

Structured illumination microscopy (SIM) goes beyond the diffraction limit by a factor of two (~100 nm lateral resolution) through illuminating the sample with structured light in such a way that fine details of the sample can later be recovered mathematically (Figure 2) [47,107]. Although SIM requires dedicated instrumentation and post-processing to generate the super-resolved image, it is amenable to standard fluorescent labels and can easily be multiplexed to image two to three targets in the same cell. SIM requires minimal modifications to sample preparation compared with confocal imaging and thus can be readily applied to *in vivo* samples. For example, 3D-SIM has been used to identify local chromatin organization around nuclear pore complexes in cultured mouse myoblasts [108]. Consistent with the observation of nucleosome clutches by STORM [79], 3D-SIM has also identified local chromatin domains on the order of 200–300 nm in cultured mouse epithelial cells [109], nanocompartments associated with TADs in cultured *Drosophila* cells and embryos [110], and similar TAD-associated nanodomains in mouse embryonic neural progenitor cells and cultured embryonic stem cells [111]. SIM has also been implemented with an all-optical approach known as instant SIM (iSIM), which dramatically increases imaging speed and requires no post-processing [112]. iSIM has been used to assess how differential organization of Pol II clusters relates to their function in both live and fixed zebrafish embryos [108,109]. Similarly, super-resolution via optical reassignment (SoRa), an implementation of iSIM that uses a spinning disk, has enabled analysis of mitotic divisions in mouse zygotes [113,114]. These examples show that SIM has great promise for imaging the nucleus *in vivo* given that pre-established labeling strategies and sample preparation techniques can be directly applied.

### Stimulated emission depletion

Stimulated Emission Depletion (STED) typically offers a ~5-fold increase in resolution over conventional microscopy, achieving lateral resolution of 50 nm or more while maintaining optical sectioning and point scanning features associated with confocal microscopy (Figure 2) [46,115,116]. STED can be readily applied to various *in vivo* systems to observe fine-scale organization within the nucleus, in part because it is largely compatible with standard sample preparation methods and shares many aspects with confocal microscopy. Although STED requires specific dyes due to the high laser powers used and necessity for spectral overlap with the depletion laser, it has been used to assess chromatin organization in cultured cells and *in vivo*. In cultured mouse embryonic stem cells, STED has been used to visualize nanoscale CTCF cluster formation and its relationship to transcription [117]. Likewise, in cultured mouse embryonic fibroblasts, STED uncovered the internal structure of chromocenters, which are too small to be resolved by diffraction-limited imaging, based on HP1 and H3K9me3 organization [118]. In developing zebrafish embryos, STED has uncovered diverse functional organizations of Pol II clusters and how nascent RNAs influence nanoscale chromatin packing [119–121]. STED has also been used to identify TF localization within individual nuclei in developing *Drosophila* [122]. In skin tissue from adult mice, 3D-STED has been used to identify compacted chromatin regions within epithelial nuclei [123].

### Expansion microscopy

As an alternative to developing and implementing complex optical setups to achieve super-resolution, expansion microscopy (ExM) instead focuses on altering the biological sample itself [124]. Samples are embedded in a swellable hydrogel that physically expands the sample upon the addition of water. By physically enlarging the sample, the distance between nearby crowded objects within the cell (e.g., proteins of interest, fluorophores, etc.) is increased according to the expansion factor, providing an increase in resolution that is proportional to the linear expansion factor. Through repeated rounds of gel-embedding and expansion, iterative ExM can reach up to 24-fold linear expansion [125–128]. This corresponds to <20 nm resolution in the imaging plane on a confocal microscope and provides an attractive option for resolving nanoscale organization within the nucleus on standard microscopes. Importantly, given the increased size of expanded samples, care must be taken to balance the numerical aperture and working distance of the imaging objective to achieve optimal images.

Because ExM alters the sample itself, there has been a strong focus on assessing and minimizing potential distortions within the cell. For example, initial studies focused on measuring distortions at the level of whole cells by mapping the expanded image back to the same cell in its unexpanded state (using a vector distortion field) and found minimal perturbations at this scale [124,125]. However, it later became apparent that even within the same cell, different organelles expanded to slightly different extents [127]. Thus, for using ExM to study nanoscale nuclear organization, it is critical to assess the expansion factor and isotropy of the nucleus itself. Initial work suggested that regions within the nucleus with different chromatin compaction states expanded differentially, leading to irregular distortions in nuclear organization [129]. Subsequently, it became clear that nuclear organization can in fact be preserved in ExM and the discrepancies between different studies may be due to different expansion protocols, fixation approaches, and labeling strategies. Faulkner et al. demonstrated the preservation of general nuclear shape with four-fold expansion in cultured human osteosarcoma cells by comparing pre- and post-expansion images of the same nuclei [130]. However, this approach is fundamentally limited by the resolution of the pre-expansion image that is used to map sample distortions, raising the possibility that nanoscale structures are perhaps perturbed.

To address this, chromatin expansion microscopy (ChromExM) was developed as an *in vivo* ExM approach specifically to image nanoscale nuclear organization (Figure 2) [131]. Using ChromExM, the authors show that organization within the nucleus is preserved during 15-fold expansion in developing zebrafish embryos by introducing fiducial markers into the DNA itself before expansion. Additionally, ChromExM also preserves the structure of *in vitro* assembled nucleosome arrays at the level of individual fibers and nucleosomes, generating images comparable to EM. ChromExM highlights the possibilities of *in vivo* analysis of nanoscale nuclear organization and function, reaching single-molecule resolution and multi-color imaging on commercially available microscopes. ChromExM was used to visualize how TFs are associated with individual nucleosomes *in vivo*, how Pol II is organized as it transcribes a specific gene through combination with RNA-FISH, and led to new insights into enhancer–promoter interactions during zygotic genome activation. Methods such as this highlight the power of ExM to enable new discoveries in nanoscale nuclear organization *in vivo* and overcome technical limitations posed by other super-resolution methods.

In cell culture, additional ExM methods have been applied to assess the localization of histone modifications and their readers [132,133]. Other related methods, such as Magnify, can achieve 11-fold expansion with just one round of gel embedding and have been used to map the localization of specific genes through combination with DNA-FISH in both human tissue sections and cultured cells [134]. Continued work to combine sequence-specific labeling of DNA and RNA with protein labeling at high expansion factors will lead to new discoveries of nuclear function at the nanoscale.

It should also be noted that ExM can be combined with other super-resolution imaging methods to further increase the resolution. For example, ChromExM has been combined with STED to image nucleosomes at ~3 nm lateral resolution; label-retention ExM has been combined with STORM and SIM to reach <10 nm resolution of clathrin-coated pits; and magnify has been combined with super-resolution optical fluctuation imaging (SOFI) to reach < 20 nm resolution to visualize basal body ultrastructure [131,133,134]. Among other examples, ONE microscopy combines 10-fold ExM with super-resolution radial fluctuation (SRRF) imaging to achieve <1 nm resolution and resolve the structure of individual proteins *in vitro* [135]. This technology moves towards structural biology by light microscopy and will likely be able to address many open questions in the nucleus.

### Image analysis

The rich datasets generated by the technologies presented here provide new challenges for image analysis yet open exciting possibilities to gain novel insights into nuclear organization and function. Generally, analysis approaches aim to delineate objects of interest (e.g., individual molecules or complexes, labeled foci marking genomic regions of interest, etc.) through segmentation. After identifying these objects, their characteristics (e.g., abundance, size, spatial distribution, etc.) and relationships to other objects are measured. These workflows have been reviewed in detail previously [136–138]. Given the complexity of multimodal 3D images of the nucleus, particularly those with single-molecule resolution, there is a need for increasingly complex analyses. These needs are being met in part with new machine-learning tools that can bypass some fallbacks of manual or fully parameterized analyses. For example, machine learning image analysis tools enable users to train custom models to identify structures of interest that may not be identified by simple intensity thresholding or identify unique spatial patterns [52]. Some widely used tools include Trainable Weka Segmentation [139], ilastik [140], and CellProfiler [141,142], among many others reviewed elsewhere [143]. As this area continues to grow, pooling resources in shared image and model repositories, such as the Image Data Resource [144], OMERO [145], the BioImage Archive [146], and the BioImage Model Zoo [147] will enable more robust training and validation of new analysis tools on a wider variety of datasets. This area is poised for continued rapid developments in coming years, such as more accessible and open source tools [148,149], to keep pace with the complex datasets being generated and will be an absolute necessity to fully understand and interpret super-resolution images of the nucleus.

## Conclusion and outlook

Over recent years, continued advances in light and electron microscopy have enabled new insights into nanoscale nuclear organization through a variety of direct visualization approaches. Key challenges that remain are adapting methods for *in vivo* applications (e.g., imaging developing and living organisms as opposed to cultured cell lines), developing diverse labeling strategies compatible with imaging at the highest resolution, and extending multiplexing capabilities to begin to unravel the complex molecular organization within the nucleus. Additionally, bridging the gap between live imaging of the highly dynamic interactions in the nucleus and fixed imaging at even higher resolution will provide a deeper understanding of nuclear function. It is likely that solutions to these challenges may soon arise from unique combinations of existing tools and techniques as has occurred over recent years with methods such as ChromExM, Magnify, MINFLUX [150,151], and ONE Microscopy. Altogether, these efforts will offer key insights into how organization within the nucleus relates to function across a variety of biological systems and contexts.

**Perspective**

- The nucleus is organized across a range of scales and requires an array of techniques to assess this organization.
- Both super-resolution light microscopy and (cryo-)EM are making advances toward visualizing and measuring nanoscale organization within the nucleus *in situ.*
- New methods that improve optical resolution and labeling capabilities for *in vivo* systems will provide new insights into nuclear organization and function.

## Abbreviations

CLEM: correlated light and electron microscopy
ChromEMT: chromatin electron microscopy with tomography
ChromSTEM: chromatin scanning transmission electron microscopy
Cryo-EM: cryo-electron microscopy
Cryo-ET: cryo-electron tomography
DNA-MERFISH: DNA multiplexed error robust FISH
DNA-PAINT: DNA-points accumulation for imaging in nanoscale topography
EM: electron microscopy
FIB-SEM: focused ion beam scanning electron microscopy
FISH: fluorescence *in situ* hybridization
Hi-C: high-throughput chromosome conformation capture
Hi-M: multiplexed sequential imaging approach
ORCA: optical reconstruction of chromatin architecture
Pol II: RNA Polymerase II
SBF-SEM: serial block-face scanning electron microscopy
SIM: structured illumination microscopy
SMLM: single-molecule localization microscopy
SOFI: super-resolution optical fluctuation imaging
SRRF: super-resolution radial fluctuation
STED: stimulated emission depletion microscop
STORM: stochastic optical reconstruction microscopy
SoRa: super-resolution via optical reassignment
TAD: topologically associating domains
TFs: transcription factors
iSIM: instant structured illumination microscopy
